# Renoprotective effects of ferric citrate in a mouse model of chronic kidney disease

**DOI:** 10.1038/s41598-022-10842-4

**Published:** 2022-04-23

**Authors:** Mark R. Hanudel, Brian Czaya, Shirley Wong, Grace Jung, Kristine Chua, Bo Qiao, Victoria Gabayan, Tomas Ganz

**Affiliations:** 1 Division of Pediatric Nephrology, Department of Pediatrics, David Geffen School of Medicine at UCLA, 10833 Le Conte Avenue, MDCC A2-383, Los Angeles, CA 90095-1752 USA; 2 Department of Medicine, David Geffen School of Medicine at UCLA, Los Angeles, CA USA

**Keywords:** Kidney diseases, Chronic kidney disease

## Abstract

In chronic kidney disease, ferric citrate has been shown to be an effective phosphate binder and source of enteral iron; however, the effects of ferric citrate on the kidney have been less well-studied. Here, in *Col4α3* knockout mice—a murine model of progressive chronic kidney disease, we evaluated the effects of five weeks of 1% ferric citrate dietary supplementation. As expected, ferric citrate lowered serum phosphate concentrations and increased serum iron levels in the *Col4α3* knockout mice. Consistent with decreased enteral phosphate absorption and possibly improved iron status, ferric citrate greatly reduced circulating fibroblast growth factor 23 levels. Interestingly, ferric citrate also lessened systemic inflammation, improved kidney function, reduced albuminuria, and decreased kidney inflammation and fibrosis, suggesting renoprotective effects of ferric citrate in the setting of chronic kidney disease. The factors mediating possible ferric citrate renoprotection, the mechanisms by which they may act, and whether ferric citrate affects chronic kidney disease progression in humans deserves further study.

## Introduction

Ferric citrate (FC) (Auryxia, Akebia Therapeutics, Inc., Cambridge, MA) is a novel compound that functions as an enteral phosphate binder and iron replacement product^1^. In randomized, placebo-controlled trials conducted in chronic kidney disease (CKD) patients not on dialysis, FC significantly decreased serum phosphate concentrations and improved iron status^2–4^. Similarly, in randomized, active-controlled trials conducted in dialysis-dependent CKD patients, FC treatment resulted in equivalent reductions in serum phosphate concentrations and improved iron status^5,6^.

In the randomized controlled trials conducted in non-dialysis-dependent CKD patients, FC also significantly reduced circulating concentrations of fibroblast growth factor 23 (FGF23)^2–4^. FGF23 is a hormone that responds to phosphate loading by reducing urinary phosphate reabsorption^7,8^ and decreasing renal 1,25-dihydroxyvitamin D production^9–11^, resulting in decreased serum phosphate concentrations. In CKD, with phosphate retention, circulating FGF23 levels increase early and continue to rise as kidney function worsens^12–16^. Although progressively increasing FGF23 concentrations in CKD help to mitigate hyperphosphatemia, elevated FGF23 levels have been independently associated with a multitude of adverse “off-target” effects, including faster CKD progression^17–19^. As both dietary phosphate absorption^20,21^ and iron deficiency^22–25^ potently stimulate FGF23 production, FC treatment targets both factors to lower circulating FGF23 levels.

In a more recent study, Block et al. conducted a 36-week randomized trial of FC vs. usual care in 199 patients with advanced CKD (estimated glomerular filtration rate < 20 ml/min/1.73m^2^)^26^. Compared to usual care, FC significantly decreased serum phosphate concentrations, increased iron parameters, and decreased circulating FGF23 levels. Interestingly, the FC-treated group had a lower incidence of a composite endpoint that included death, provision of dialysis, or transplantation, suggesting possible beneficial effects on the kidney. The results from this trial are consistent with pre-clinical in vivo data from a study by Francis et al. in which *Col4α3* knockout mice (a murine model of Alport syndrome CKD) were treated with a 5% FC-supplemented diet^27^. The FC-treated mice had decreased serum phosphate concentrations, increased iron parameters, and decreased circulating FGF23 levels, as well as improved kidney function and markers of fibrosis. As a 5% FC diet is a high dose of the drug, we evaluated the effects of 1% FC treatment in the *Col4α3* knockout murine model of progressive CKD. As detailed later in the manuscript, a 1% FC diet may be more similar to human doses of the drug. In this CKD model, we assessed parameters related to mineral metabolism, iron metabolism, erythropoiesis, inflammation, and kidney function and fibrosis.

## Results

### Effects of ferric citrate on mineral metabolism parameters

**Figure 1:**
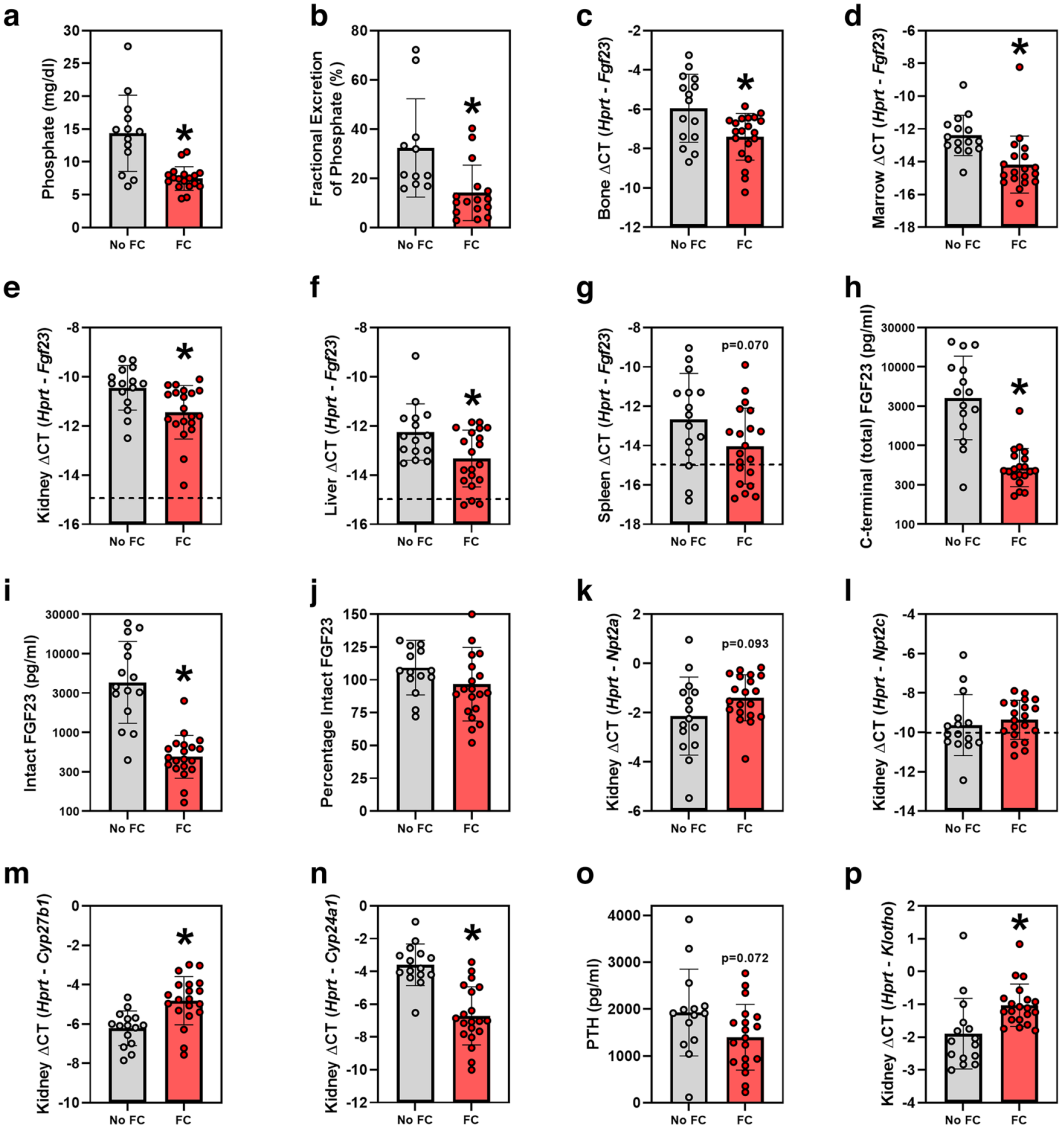
Mineral metabolism parameters in *Col4α3* knockout mice treated with or without 1% ferric citrate (FC) added to the diet. Parameters measured include: (**a**) serum phosphate, (**b**) urinary fractional excretion of phosphate, (**c**) bone *Fgf23* mRNA, (**d**) marrow *Fgf23* mRNA, (**e**) kidney *Fgf23* mRNA, (**f**) liver *Fgf23* mRNA, (**g**) spleen *Fgf23* mRNA, (**h**) plasma C-terminal (total) FGF23, (**i**) plasma intact FGF23, (**j**) plasma percentage intact FGF23, (**k**) kidney *Npt2a* mRNA, (**l**) kidney *Npt2c* mRNA, (**m**) kidney *Cyp27b1* mRNA, (**n**) kidney *Cyp24a1* mRNA, (**o**) plasma parathyroid hormone, and (**p**) kidney *Klotho* mRNA. In figures (**e**), (**f**), (**g**), and (**l**), dotted lines correspond to quantitative real-time PCR cycle threshold values for blank samples. Data are presented as means and standard deviations. Two-tailed t-tests are used to compare groups, *p < 0.05.

In our *Col4α3* knockout mice, five weeks of 1% FC dietary supplementation significantly decreased serum phosphate concentrations by 48% (mean (SD) 7.5 (1.8) vs. 14.3 (5.8) mg/dl, *p* < 0.001; Fig. 1a). Consistent with lower serum phosphate levels, the urinary fractional excretion of phosphate was decreased in FC-treated mice (Fig. 1b). FC treatment significantly decreased bone, marrow, kidney, and liver *Fgf23* mRNA expression, and tended to decrease spleen *Fgf23* mRNA expression (Fig. 1c–g). Circulating concentrations of C-terminal (total) FGF23 (Fig. 1h) and intact FGF23 (Fig. 1i) were decreased by an order of magnitude in FC-treated mice (total FGF23 geometric mean (95% CI) 526 (412, 672) vs. 4018 (2162, 7470) pg/ml, *p* < 0.001; intact FGF23 geometric mean (95% CI) 490 (372, 644) vs. 4319 (2354, 1923) pg/ml, *p* < 0.001). In both groups, almost all of the circulating FGF23 was intact (Fig. 1j). Consistent with decreased FGF23 effects, kidney type 2a sodium-phosphate cotransporter (*Npt2a*) mRNA expression tended to increase; kidney 1α-hydroxylase (*Cyp27b1*) mRNA expression increased; and kidney 24-hydroxylase (*Cyp24a1*) mRNA expression decreased; kidney type 2c sodium-phosphate cotransporter (*Npt2c*) mRNA expression did not significantly increase (Fig. 1k–n). Circulating PTH concentrations tended to be lower in FC-treated mice (mean (SD) 1399 (700) vs. 1925 (926) pg/ml, *p* = 0.072; Fig. 1o). Lastly, FC treatment significantly increased kidney *Klotho* mRNA expression (Fig. 1p).

### Effects of ferric citrate on iron-related and erythropoietic parameters

**Figure 2:**
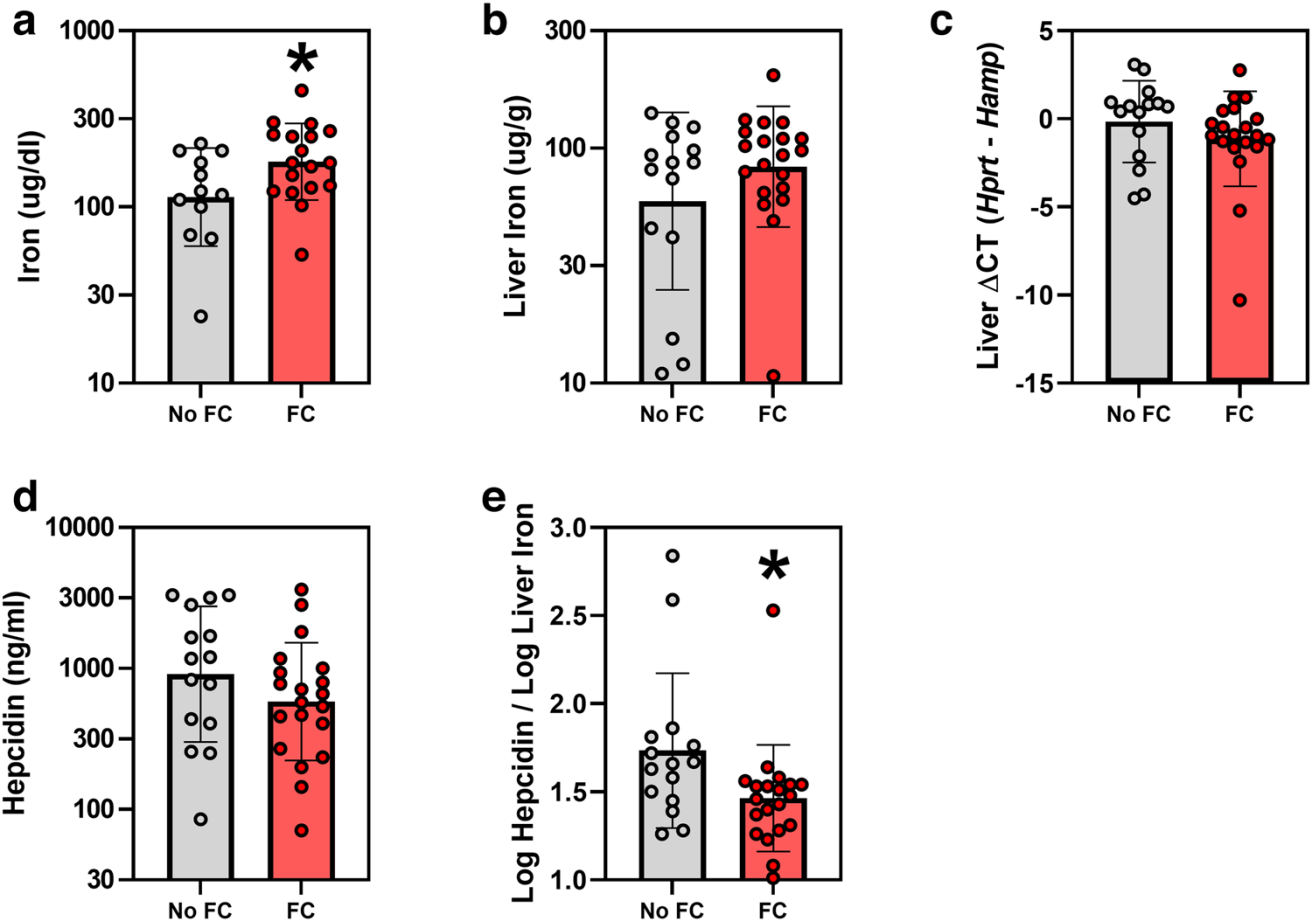
Iron parameters in *Col4α3* knockout mice treated with or without 1% ferric citrate (FC) added to the diet. Parameters measured include: (**a**) serum iron, (**b**) liver iron, (**c**) liver *Hamp* mRNA, (**d**) serum hepcidin, and (**e**) log serum hepcidin / log liver iron. Data are presented as means and standard deviations. Two-tailed t-tests are used to compare groups, *p < 0.05.

**Figure 3:**
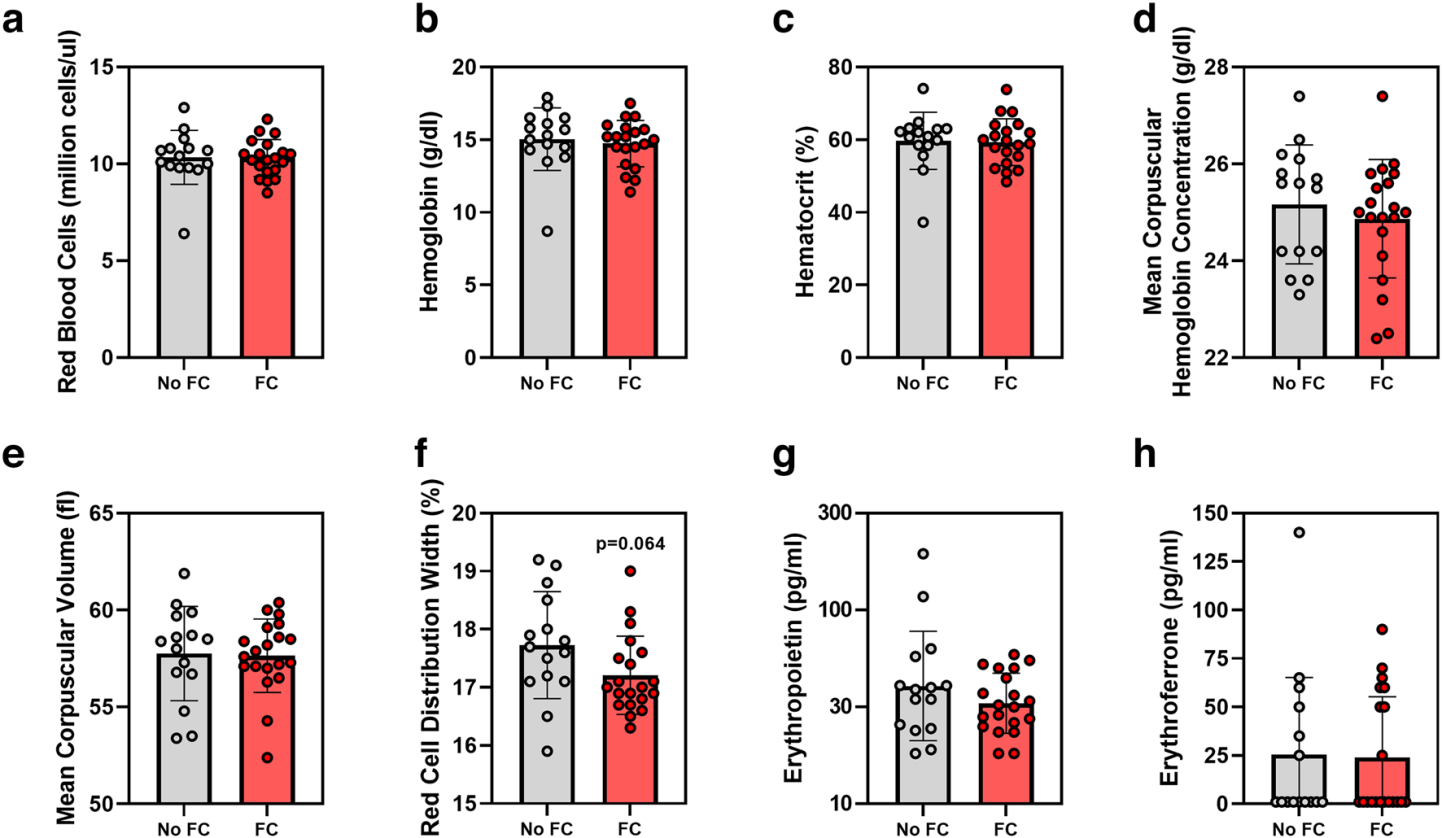
Erythropoietic parameters in *Col4α3* knockout mice treated with or without 1% ferric citrate (FC) added to the diet. Parameters measured include: (**a**) red blood cells, (**b**) hemoglobin, (**c**) hematocrit, (**d**) mean corpuscular hemoglobin concentration, (**e**) mean corpuscular volume, (**f**) red cell distribution width, (**g**) serum erythropoietin, and (**h**) serum erythroferrone. Data are presented as means and standard deviations. Two-tailed t-tests are used to compare groups, *p < 0.05.

FC treatment significantly increased serum iron concentrations in *Col4α3* knockout mice by 59% (geometric mean (95% CI) 180 (145, 224) vs. 113 (82, 156) ug/dl, *p* = 0.033; Fig. 2a). FC-induced absolute changes in liver iron did not reach statistical significance (geometric mean (95% CI) 83 (64, 108) vs. 59 (38, 92) ug/g, p = 0.18; Fig. 2b). Despite iron loading with FC, liver hepcidin (*Hamp*) mRNA did not increase (Fig. 2c), and serum hepcidin concentrations numerically decreased (geometric mean (95% CI) 583 (382, 889) vs. 913 (522, 1599) ng/ml, *p* = 0.21; Fig. 2d). However, the ratio of log-transformed serum hepcidin concentrations to log-transformed liver iron was significantly decreased (Fig. 2e), suggesting that, for the degree of iron loading, hepcidin levels were lower. Erythropoietic parameters, including red blood cells, hemoglobin, hematocrit, mean corpuscular hemoglobin concentration, mean corpuscular volume, red cell distribution width, serum erythropoietin, and serum erythroferrone (Fig. 3a–h) did not significantly differ between the groups.

### Effects of ferric citrate on markers of systemic and kidney inflammation

**Figure 4:**
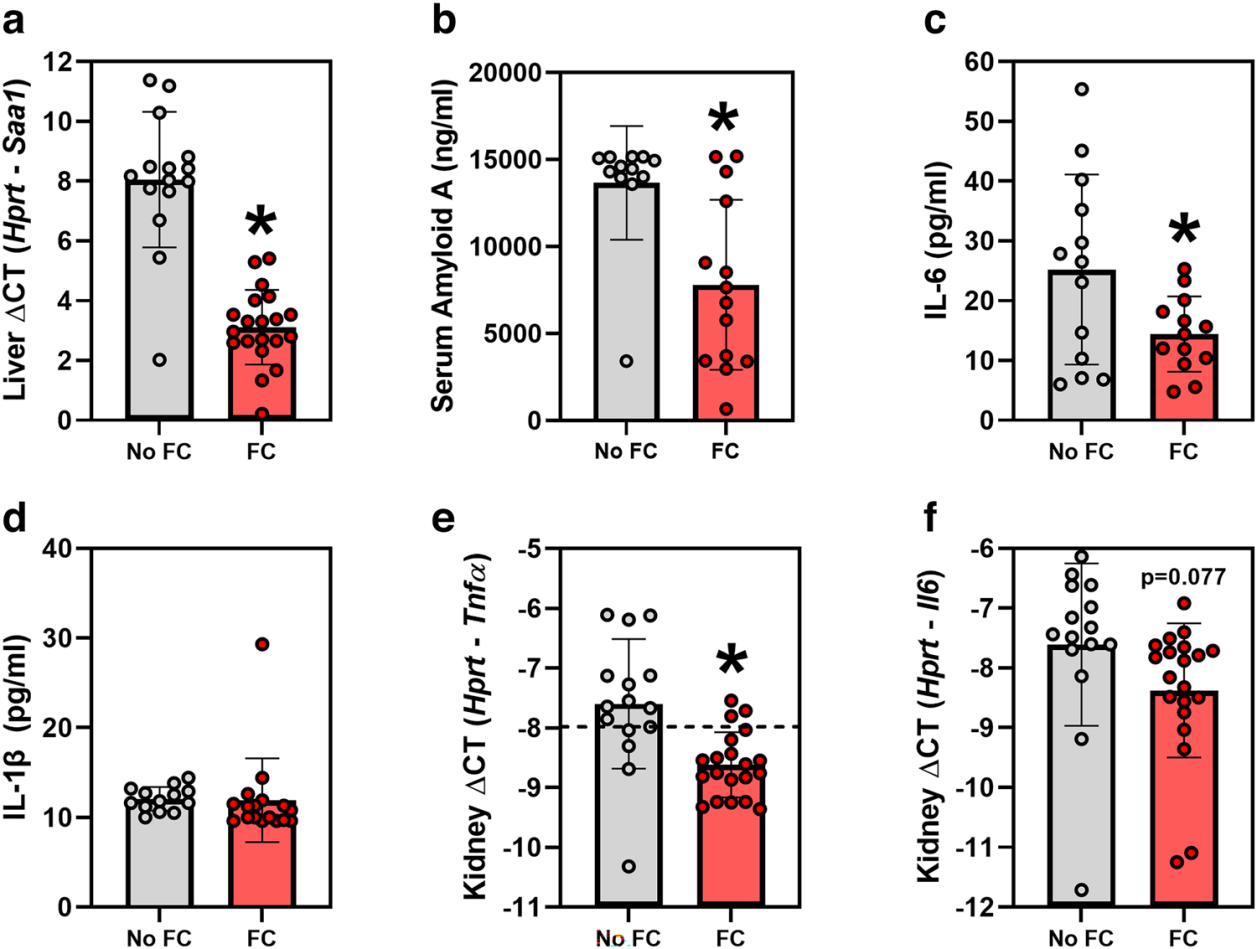
Markers of inflammation in *Col4α3* knockout mice treated with or without 1% ferric citrate (FC) added to the diet. Parameters measured include: (**a**) liver *Saa1* mRNA, (**b**) serum amyloid A, (**c**) serum interleukin 6, (**d**) serum interleukin 1β, (**e**) kidney *Tnfα* mRNA, and (**f**) kidney *Il6* mRNA. In figure (**e**), the dotted line corresponds to quantitative real-time PCR cycle threshold values for blank samples. Data are presented as means and standard deviations. Two-tailed t-tests are used to compare groups, *p < 0.05.

FC treatment significantly decreased markers of systemic inflammation in *Col4α3* knockout mice, including liver *Saa1* mRNA (Fig. 4a), serum amyloid A concentrations (Fig. 4b), and serum IL-6 concentrations (Fig. 4c). Serum IL-1β concentrations did not differ between the groups (Fig. 4d). In the kidney, FC treatment significantly decreased *Tnfα* mRNA expression (Fig. 4e) and tended to decrease *Il6* mRNA expression (Fig. 4f).

### Effects of ferric citrate on kidney function and fibrosis

**Figure 5:**
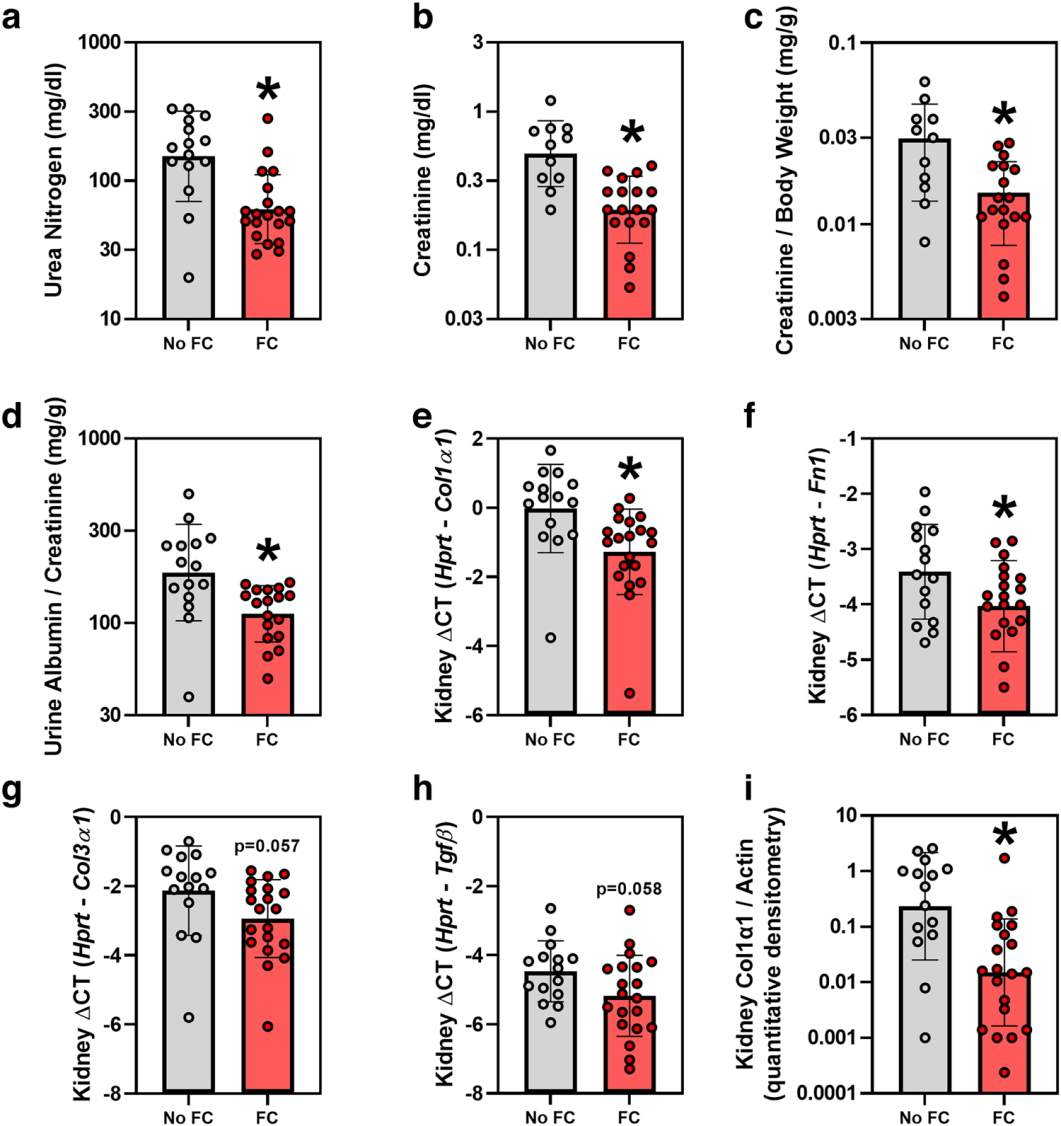
Kidney function parameters and markers of fibrosis in *Col4α3* knockout mice treated with or without 1% ferric citrate (FC) added to the diet. Parameters measured include: (**a**) serum urea nitrogen, (**b**) serum creatinine, (**c**) serum creatinine normalized to body weight, (**d**) urine albumin-to-creatinine ratio, (**e**) kidney *Col1α1* mRNA, (**f**) kidney fibronectin (*Fn1*) mRNA, (**g**) kidney *Col3α1* mRNA, (**h**) kidney *Tgfβ* mRNA, and (**i**) kidney Col1α1 protein, assessed via Western blot quantitative densitometry. Data are presented as means and standard deviations. Two-tailed t-tests are used to compare groups, *p < 0.05.

In this murine CKD model, FC treatment significantly decreased serum urea nitrogen concentrations (geometric mean (95% CI) 62 (48, 80) vs. 150 (103, 220) mg/dl, *p* < 0.001 (59% reduction); Fig. 5a); serum creatinine (geometric mean (95% CI) 0.20 (0.15, 0.25) vs. 0.50 (0.38, 0.66) mg/dl, *p* < 0.001 (60% reduction); Fig. 5b); serum creatinine normalized to body weight (Fig. 5c); and the urine albumin-to-creatinine ratio (geometric mean (95% CI) 112 (96, 131) vs. 188 (138, 255) mg/g, p = 0.005 (40% reduction); Fig. 5d). FC treatment also significantly decreased kidney mRNA expression of *Col1α1* (Fig. 5e) and fibronectin (*Fn1*; Fig. 5f), and tended to decrease kidney mRNA expression of *Col3α1* (Fig. 5g) and *Tgfβ* (Fig. 5h). FC treatment significantly decreased the amount of Col1α1 protein in the kidney (Fig. 5i; Fig. S1).

## Discussion

In randomized controlled trials conducted in patients with CKD, ferric citrate (FC) has been shown to control serum phosphate, improve iron status, and decrease circulating FGF23 concentrations^2–6,26^, with one small trial observing improved clinical outcomes with FC treatment^26^. How FC treatment may affect renal parameters, including kidney disease progression, in patients with CKD is unknown. In the present study, we treated *Col4α3* knockout mice (a murine model of progressive CKD) with a diet supplemented with 1% FC, a lower dose than has been previously assessed in rodent CKD models^27,28^. Compared to untreated *Col4α3* knockout mice, the FC-treated mice had decreased serum phosphate concentrations, increased serum iron, dramatically reduced circulating FGF23 levels, decreased markers of systemic and kidney inflammation, improved kidney function, and decreased kidney fibrosis (as evidenced by decreased collagen type I accumulation in the kidney^29^). Our results are consistent with those reported by Francis et al., where *Col4α3* knockout mice were treated with a 5% FC diet^27^. Improved kidney function and decreased expression of pro-fibrotic molecules was also observed in a study of 5/6 nephrectomy CKD rats treated with a 4% FC diet^28^.

In our murine CKD model, the beneficial effects of FC treatment on kidney function, markers of inflammation, and markers of fibrosis are intriguing. The underlying mechanisms by which FC may have renoprotective effects are unclear and may be multiple. It is possible that FC-induced lowering of serum phosphate and/or FGF23 concentrations may have favorable effects on the diseased kidney. Several rodent CKD studies have assessed the effects of low or high phosphate diets on the kidney. Uremic mice and rats fed low phosphate diets have improved kidney function and histology^30–32^, consistent with human CKD studies^30,33^. Conversely, CKD^31^ and non-CKD^34^ rodents fed high phosphate diets develop severe renal fibrosis.

However, given that in vivo animal studies of the effects of phosphate rely on the administration of high or low phosphate diets, they may be confounded by the expected increase or decrease in FGF23 concentrations that occurs with enteral phosphate loading or restriction. Yet, there is some evidence of FGF23-independent effects of phosphate on the kidney. In a study of *Col4*α*3* knockout mice fed a low (0.2%) or normal (0.54%) phosphate diet, the CKD mice on the low phosphate diet had lower serum phosphate and creatinine, but similar FGF23 levels^32^, suggesting that changes in phosphate rather than FGF23 affected kidney function. In another study, non-CKD mice received a single injection of sodium phosphate or recombinant FGF23^27^. Two hours post-injection, sodium phosphate, but not recombinant FGF23, increased renal mRNA expression of fibrosis markers *Col1*α*1*, *Col3α1*, and *Col6α1*. These data suggest that high phosphate alone, independently of FGF23, may promote renal fibrosis. Lastly, in a murine CKD model with or without *Fgf23* deletion, the mice lacking *Fgf23* developed more pronounced hyperphosphatemia, worse kidney function, and increased kidney fibrosis, suggesting FGF23-independent adverse effects of high phosphate concentrations on the diseased kidney^35^.

Mechanisms by which elevated phosphate concentrations may promote kidney dysfunction and fibrosis include luminal precipitation of calcium phosphate crystals, which damage tubular cells^36^; direct increase of fibronectin expression, which has been observed in vitro^37^; and possibly endothelial dysfunction^38^. Additionally, as high phosphate levels may be pro-inflammatory^31,39,40^, potentially mediated by NF-κB signaling pathways^41,42^, it is possible that changes in inflammation^43^ may mediate an indirect relationship between phosphate and fibrosis.

Like high phosphate concentrations, increased FGF23 levels may also have detrimental effects on diseased kidneys. In multiple CKD cohorts^17–19^, higher FGF23 levels are independently associated with faster CKD progression. Higher circulating FGF23 concentrations may promote CKD progression via activation of pro-fibrotic renal transforming growth factor beta (TGF-β) signaling pathways^44–46^. Additionally, FGF23 may promote inflammation in CKD^47^, which may contribute to the development of fibrosis^43^.

Thus, it may be hypothesized that lowering FGF23 concentrations to some extent may have beneficial effects in CKD. FC decreases FGF23 levels by targeting two important stimuli of FGF23 production, enteral phosphate absorption^20,21^ and iron deficiency^22–25^. In our murine CKD model, FC treatment decreased serum phosphate by 48% and increased serum iron by 59%, changes that likely contributed to the substantial order-of-magnitude reduction in circulating FGF23 concentrations. FGF23 is known to decrease expression of type 2a and 2c sodium-phosphate cotransporters in the renal proximal tubules^7,8^, decrease expression of renal 1α-hydroxylase, and increase expression of renal 24-hydroxylase^9–11^. In our study, the observed downstream effects on *Npt2a*, *Cyp27b1*, and *Cyp24a1* mRNA expression in FC-treated mice are consistent with reduced effects of circulating FGF23. Although FGF23 is thought to be a predominantly bone-derived hormone, several other organs and tissues are capable of increased *Fgf23* mRNA expression, including the marrow^48–50^, kidney^51–53^, liver^54,55^, and spleen^54,56–58^. Interestingly, FC treatment decreased *Fgf23* mRNA expression in multiple organs and tissues, including the bone, marrow, kidney, liver, and spleen, suggesting that FC treatment may affect both osseous and extra-osseous FGF23 production.

The effects of iron deficiency on FGF23 production and metabolism differ in the presence or absence of CKD. In non-CKD models, iron deficiency concurrently increases *Fgf23* mRNA transcription and FGF23 post-translational proteolytic cleavage, resulting in cellular secretion of large quantities of FGF23 protein fragments^22–25^. However, CKD is a state of impaired FGF23 proteolytic cleavage^59–61^, lessening FGF23 fragment production. Indeed, in a study of mice with and without CKD, an iron deficient diet increased whole bone *Fgf23* mRNA expression and plasma concentrations of total (intact + fragmented) FGF23 to similar degrees, but increased plasma concentrations of full-length intact FGF23 to a much greater extent in the mice with CKD^25^. Whereas the median percentage of circulating FGF23 that was intact was only 12% in the non-CKD iron-deficient mice, it was 72% in the CKD iron-deficient group^25^.

In our model, FC treatment increased serum iron concentrations. We also observed numeric increases in liver iron and decreases in serum hepcidin, but these changes did not reach statistical significance. However, the ratio of serum hepcidin to liver iron was significantly decreased, suggesting that, for the degree of iron loading, hepcidin levels were lower. Hepcidin production is induced by iron loading^62^ and inflammation^63^, and kidney excretion represents a major mode of hepcidin clearance^64^. Therefore, in our CKD model, the decreased serum hepcidin concentrations, despite increased iron levels, may be explained by less systemic inflammation, specifically decreased IL-6^65^, and/or improved kidney function. Lastly, our mice did not become anemic and, despite increased serum iron levels, FC treatment did not affect hemoglobin concentrations.

How optimization of iron status per se in CKD affects disease progression is unclear. Whereas anemia may be a risk factor for progression of CKD^66,67^, the role of iron status in CKD is not well-defined. In a large, recent study of patients with moderate CKD, it was observed that, as compared to anemia without iron deficiency, neither absolute iron deficiency anemia nor functional iron deficiency anemia were significantly associated with dialysis initiation^68^. In another recent study of CKD patients, higher serum concentrations of non-transferrin bound iron (NTBI) were independently associated with a composite kidney endpoint that included renal replacement therapy, a 40% decline in estimated glomerular filtration rate, or death due to kidney disease^69^, likely mediated by the pro-inflammatory and/or pro-oxidative effects of NTBI. In pre-clinical CKD models, differential effects of iron therapy on kidney function and fibrosis have been observed, depending on the specific iron preparation and the route of administration^70^. For instance, in one study of mice with adenine diet-induced CKD, oral carbonyl iron worsened kidney function, but intraperitoneal iron dextran improved kidney function, a difference possibly contributed to by the systemic pro-inflammatory effect of chronic oral carbonyl iron administration^71^.

In this study, we fed CKD mice diets containing 1% FC, which is a lower dose than that used in previously published rodent CKD studies^27,28^. The bioequivalence of this dose in humans can be considered as follows. For the enteral phosphate binding effects of FC, drug activity depends on intestinal intraluminal concentration. The 129 mouse strains consume approximately 4 g of dry food and 5 ml of water (1 g/milliliter density) daily^72^, for a total of 9 g of dry food + water consumed daily. Therefore, dry food constitutes 4 g / 9 g = 0.44 (44%) of the daily intestinal intraluminal content; with dry food that is 1% FC, the functional intestinal intraluminal concentration of FC is 0.44 × 1% = 0.44%. Assuming humans consume approximately 2500 kcal daily (~ 33 kcal/kg/day in a 75 kg adult), at an average caloric value of 5 kcal/gram, this would represent 500 g of food daily. Daily fluid consumption of approximately 2 L would give a total of approximately 2500 g of food + fluid consumed daily (although this value would likely be less in fluid-restricted dialysis patients). Dosing FC at 6–12 g daily would result in a functional intestinal intraluminal concentration of 6–12 g / 2500 g = 0.24–0.48%. Thus, for enteral phosphate binding, the FC dose used in this mouse study may be considered reasonably similar to human dosing.

In summary, in the *Col4α3* knockout murine model of CKD, we observed that treatment with 1% FC—a lower dose than previously reported—decreased serum phosphate concentrations, increased iron parameters, substantially reduced circulating FGF23 levels, decreased markers of systemic and kidney inflammation, improved kidney function, and decreased kidney fibrosis. Although the *Col4α3* knockout mouse is a specific model of CKD, renoprotective effects of FC have been observed in other rodent CKD models^28^, suggesting a generalized effect. Further studies will be required to determine what specifically mediates FC-induced renoprotection—decreased phosphate, improved iron status, and/or FGF23 reduction—and whether or not these potentially contributory factors act directly or indirectly on the kidney. Regardless of the underlying mechanisms by which FC may beneficially affect the kidney, the present data support further human studies to determine whether or not FC alters CKD progression.

## Methods

### Mouse experiments

Experiments were conducted in accordance with UCLA Division of Laboratory Animal Medicine guidelines, and the study protocols were approved by the UCLA Office of Animal Research Oversight. The study is reported in accordance with the ARRIVE guidelines.

Mice were housed at UCLA, in standard cages with wood chip bedding that was changed twice weekly. Animal housing rooms were temperature and humidity controlled, with a 12-h light cycle. Five-week-old *Col4α3* knockout mice were placed on five-week diets (Envigo, Hackensack, NJ; TD.80394, 0.54% phosphate, 48 ppm iron) with (n = 20) or without (n = 15) 1% ferric citrate (FC; Akebia). Both dietary groups contained half males and half females. Mice were euthanized via isoflurane overdose at ten weeks of age. After sacrifice, we collected urine, whole blood, serum, plasma, liver, spleen, kidney, and tibias, from which we flushed the bone marrow with saline solution and 28G syringes.

### Blood and urine assays

Complete blood counts were measured by the Hemavet® 950 automated processor (Drew Scientific, Oxford, CT). Colorimetric methods were used to assay serum and urine phosphate (StanBio Labs, Boerne, TX), serum iron (Genzyme, Cambridge, MA), serum urea nitrogen (BioAssay Systems, Hayward, CA), and serum and urine creatinine (Crystal Chem, Elk Grove Village, IL). Commercially available enzyme-linked immunosorbent assays (ELISA) kits were used to measure urine albumin (Abcam, Cambridge, MA), serum erythropoietin (R&D Systems, Minneapolis, MN), serum amyloid A (R&D Systems), serum interleukin 1β (R&D Systems), serum interleukin 6 (R&D Systems), plasma parathyroid hormone (PTH) (Quidel, San Diego, CA), plasma C-terminal (total) fibroblast growth factor 23 (FGF23) (Quidel), and plasma intact FGF23 (Quidel). Whereas the total FGF23 assay detects both full-length, intact FGF23 and C-terminal FGF23 proteolytic fragments, the intact FGF23 assay detects only the full-length form. Percentage intact FGF23 was calculated as follows: ( [intact FGF23] / [total FGF23] ) × 100%. In-house ELISAs were used to measure serum hepcidin^73^ and serum erythroferrone^74^, as previously described.

### Quantitative liver iron concentrations

Harvested livers were snap-frozen in liquid nitrogen and stored at − 80 °C. Small pieces of the livers (~ 100 mg) were weighed and homogenized. Protein precipitation solution (0.53 N HCl and 5.3% trichloroacetic acid in ddH_2_O) was added, and the samples were boiled and centrifuged. Iron concentrations in the supernatants were measured by a colorimetric assay (Genzyme), then normalized to the weights of the original samples to yield tissue iron concentrations.

### Quantitative real-time PCR

Kidney, liver, spleen, flushed tibias, and isolated bone marrow were homogenized in Trizol (Invitrogen, Waltham, MA), and RNA was isolated according to the manufacturer’s protocol. We performed quantitative RT-PCR using the iScript RT-PCR kit (Bio-Rad, Hercules, CA). Mouse primer sequences are listed in the Table S1. We used the following PCR conditions: initial denaturation at 95 °C for 2 min, followed by 40 cycles of denaturation at 94 °C for 30 s, annealing at 58 °C for 30 s, extension at 72 °C for 1 min, and final extension at 72 °C for 10 min. Gene expression was normalized to that of hypoxanthine–guanine phosphoribosyltransferase (*Hprt*), and each RNA sample was analyzed in duplicate.

### Western blotting

For collagen I detection on Western blot, the primary antibody used was rabbit anti-mouse collagen I (ab21286, Abcam), at a concentration of 1:2000, and the secondary antibody used was horseradish peroxidase–labeled goat anti-rabbit antibody (31460; Thermo Fisher Scientific), at a concentration of 1:10,000. For the actin protein loading control, mouse anti-actin antibody (A3854, Sigma-Aldrich (St. Louis, MO)), at a concentration of 1:50,000, was used. Chemiluminescent signal was detected using the ChemiDoc XRS + System with Image Lab Software (Bio-Rad). Quantitative densitometry was performed, normalizing to the same sample across multiple gels.

### Statistical analysis

Figure creation and statistical analysis was performed using GraphPad Prism 9.2.0 (San Diego, CA). Data are presented as means and standard deviations (SD), and two-tailed t-tests were used to compare means between the groups, with *p* < 0.05 considered statistically significant. For non-normally distributed data, log transformation was applied prior to statistical analysis.

## Supplementary Information

Supplementary Information.

## Data Availability

All data generated or analyzed during this study are included in this published article (and its Supplementary Material files).
