# Supplementary material for: Renoprotective effects of ferric citrate in a mouse model of chronic kidney disease
Source: Sci Rep. 2022 Apr 23;12:6695. doi: 10.1038/s41598-022-10842-4 (PMC9035171; doi:10.1038/s41598-022-10842-4)
Supplement: Supplementary file 1 — Supplementary Information. [file 41598_2022_10842_MOESM1_ESM.pdf]

Renoprotective effects of ferric citrate in a mouse model of chronic kidney disease

Hanudel MR, Czaya B, Wong S, Jung G, Chua K, Qiao B, Gabayan V, Ganz T

<u>Supplementary Material</u>:

<u>Figure S1</u>: Kidney Col1α1 protein, assessed via Western blot, in *Col4α3* knockout mice treated with or without 1% ferric citrate (FC) added to the diet.

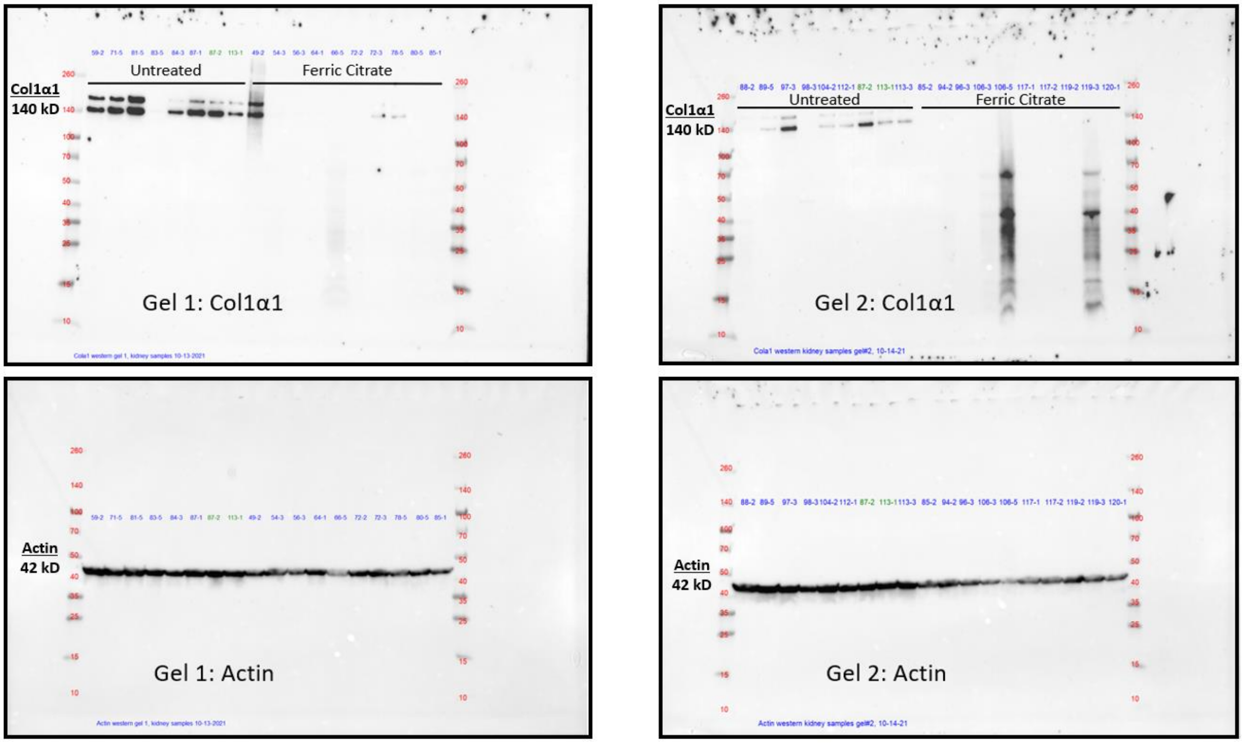

<u>Table S1</u>: Primer sequences used for quantitative real-time PCR.

**Mouse Gene of Interest**	**Primer Sequence**
Col1α1 (forward)	5'-ATGGATTCCCGTTCGAGTACG-3'
Col1α1 (reverse)	5'-TCAGCTGGATAGCGACATCG-3'
Col3α1 (forward)	5'-GACCAAAAGGTGATGCTGGACAG-3'
Col3α1 (reverse)	5'-CAAGACCTCGTGCTCCAGTTAG-3'
Cyp24a1 (forward)	5'-GCTCCTTCAAAAGGACACAGAGG-3'
Cyp24a1 (reverse)	5'-CGCTTGCCACACTTTGGTGTTG-3'
Cyp27b1 (forward)	5'-TTCGGCTTTGGCAAACGGAGCT-3'
Cyp27b1 (reverse)	5'-GGCTTGATAGGAAGAGCACCTG-3'
Fgf23 (forward)	5’-ACAGGAGCCATGACTCGAAG-3’
Fgf23 (reverse)	5’-GCAATTCTCTGGGCTGAAGT-3’
Fn1 (forward)	5'-GATGTCCGAACAGCTATTTACCA-3'
Fn1 (reverse)	5'-CCTTGCGACTTCAGCCACT-3'
Hamp (forward)	5’-TTGCGATACCAATGCAGAAGA-3’
Hamp (reverse)	5’-GATGTGGCTCTAGGCTATGTT-3’
Hprt (forward)	5’-CTGGTTAAGCAGTACAGCCCCAA-3’

Hprt (reverse)	5’-CAGGAGGTCCTTTTCACCAGC-3’
Il6 (forward)	5'-TACCACTTCACAAGTCGGAGGC-3'
Il6 (reverse)	5'-CTGCAAGTGCATCATCGTTGTTC-3'
Klotho (forward)	5'-TGATGTCGTCCAACACGTAGGCTT-3'
Klotho (reverse)	5'-GCAAAGTGCTCAACTGGCTAAGGT-3'
Saa1 (forward)	5’-AGTCTGGGGTGCTGAGAAAA-3’
Saa1 (reverse)	5’-ATGTCTGTTGGCTTCCTGGT-3’
Npt2a (forward)	5'-GGCTCCAACATTGGCACTACCA-3'
Npt2a (reverse)	5'-ACCACAGTAGGATGCCCGAGAT-3'
Npt2c (forward)	5'-GCGGTATTACCAGCAACACCAC-3'
Npt2c (reverse)	5'-TGTCCTCCTCTGGAGATGCTGA-3'
Tgfβ (forward)	5'-ATACGTCAGACATTCGGGAAGCAGTG-3'
Tgfβ (reverse)	5'-AATAGTTGGTATCCAGGGCTCTCCG-3'
Tnfα (forward)	5'-GGTGCCTATGTCTCAGCCTCTT-3'
Tnfα (reverse)	5'-GCCATAGAACTGATGAGAGGGAG-3'
